# Vesicle trafficking pathways that direct cell migration in 3D matrices and in vivo

**DOI:** 10.1111/tra.12605

**Published:** 2018-09-10

**Authors:** Beverley J. Wilson, Jennifer L. Allen, Patrick T. Caswell

**Affiliations:** ^1^ Wellcome Trust Centre for Cell‐Matrix Research, Faculty of Biology, Medicine and Health University of Manchester, Manchester Academic Health Science Centre Manchester UK

**Keywords:** cell migration, cell signalling, endocytic trafficking, invasive migration, in vivo migration

## Abstract

Cell migration is a vital process in development and disease, and while the mechanisms that control motility are relatively well understood on two‐dimensional surfaces, the control of cell migration in three dimensions (3D) and in vivo has only recently begun to be understood. Vesicle trafficking pathways have emerged as a key regulatory element in migration and invasion, with the endocytosis and recycling of cell surface cargos, including growth factor and chemokine receptors, adhesion receptors and membrane‐associated proteases, being of major importance. We highlight recent advances in our understanding of how endocytic trafficking controls the availability and local activity of these cargoes to influence the movement of cells in 3D matrix and in developing organisms. In particular, we discuss how endocytic trafficking of different receptor classes spatially restricts signals and activity, usually to the leading edge of invasive cells.

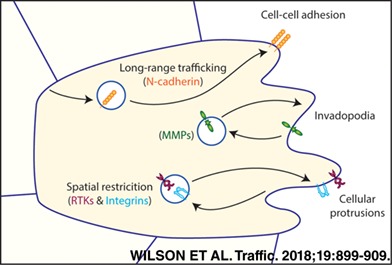

## 1 INTRODUCTION

Cell migration is a fundamental physiological process, which is essential to tissue homeostasis and wound healing, as well as gastrulation and organ generation during embryonic development. Abnormal cell migration is known to play a major role in a range of pathological conditions, including cancer metastasis, atherosclerosis and inflammation. As such, a complete understanding of the complex mechanisms that drive cell migratory processes should enable therapeutic manipulation.

Accumulating evidence demonstrates a role for membrane trafficking in the regulation of cell migration in a variety of contexts. Many studies have shown that internalization and recycling of adhesion receptors to be particularly important, for example, integrins and syndecans, which regulate cell adhesion to the extracellular matrix (ECM),^1^ and cadherins, which regulate cell‐to‐cell adhesion.^2^

There is also extensive evidence that polarized recycling of receptor tyrosine kinases (RTKs) can generate localized signalling at the leading edge in response to extracellular signals or chemokine gradients, stimulating cell migration in a directional manner.^1^ These endosomal trafficking pathways ultimately activate signalling cascades, thereby enabling the reorganization of the actin cytoskeleton and cell migration.

Increasing evidence also implicates Golgi orientation to the leading edge, Golgi morphology and the polarization of post‐Golgi anterograde transport in the regulation of cell motility, particularly in two dimensions (2D).^3^, ^4^, ^5^, ^6^ Although trafficking from the Golgi is of importance in cell migration regulation, this review will instead focus on the role of endosomal receptor recycling in this process.

Study of cell migration in simple two‐dimensional (2D) environments has led to the detailed characterization of lamellipodial migration, which is driven by fan‐like actin‐rich membrane protrusions at the leading edge.^7^ However, 2D surfaces do not accurately represent three‐dimensional (3D) in vivo environments, in which cells must navigate through a plethora of obstacles, including ECM, other cells and tissue boundaries. Recent research has shifted towards investigating cell migration within environments that more closely recapitulate those present in vivo, allowing the characterization of a range of 3D migratory modes, including mesenchymal, amoeboid and lobopodial^8^ (Box 1). Here, we highlight recent examples demonstrating that endosomal trafficking of cargoes, in particular adhesion receptors and RTKs, controls cell migration and invasion both in 3D microenvironments and in vivo.

BOX 1 **METHODS TO STUDY CELL MIGRATION IN 2D VS 3D MATRIX**

2D assays: Scratch wound and random migration of cells plated on plastic/glass (and matrix‐coated surfaces), captured by time lapse imaging. Advantages include ease of imaging, ability to quantify numerous parameters (eg, speed, directional persistence). The major disadvantage is the lack of physiological relevance, as plastic and glass surfaces are more rigid than surfaces found in vivo, and often cells move through fibrillar 3D matrix of interstitial tissue.^107^

Cell‐derived matrices (CDMs): Fibroblasts lay down a collagen‐ and fibronectin‐rich fibrillar matrix (resembling interstitial matrix) on tissue culture plastic before being removed leaving a 10‐20 µm thick layer of matrix behind.^108^ Cells plated on CDMs move on and in the matrix and can be imaged using time‐lapse microscopy. The major advantage is that cells move in a more physiological matrix, in which the orientation of matrix ligands and bundling of fibrillar matrix components is organized by fibroblasts. Cells generate cell‐matrix adhesion complexes^108^ (broadly similar to fibrillar adhesions) and this set up is particularly amenable to high‐resolution imaging. The disadvantage is that while cells move in and on a 3D matrix, they move in a very narrow z‐range (albeit without contacting the glass or plastic substrate).

Hydrogels: Numerous hydrogel type systems exist, from artificially fabricated systems to purified matrix proteins (matrigel, fibrillar collagen). The clear advantage here is that cells move in a defined 3D environment, and matrix components can be “tuned” to resemble specific matrices found in vivo. The major disadvantage is the difficulty in imaging cells (particularly at high resolution) and tracking cell movement in xyx planes. Also, fibrillar structures and matrix ligands are randomly organized. An alternative approach is organotypic assays, in which fibroblasts pre‐strain a 3D collagen hydrogel and reorganize the matrix to facilitate invasion of other cell types.^109^, ^110^

## 2 ENDOSOMAL TRAFFICKING

Cell surface proteins are internalized via clathrin‐mediated endocytosis (CME) or clathrin‐independent endocytosis (CIE), and cargoes are delivered to early endosomes for recycling back to the cell surface or targeting for degradation.^9^ Early endosomes undergo maturation to late endosomes, where cargoes can be compartmentalized into acidic intraluminal vesicles. Cargoes confined to vesicles are dissociated from ligands and degraded upon fusion of late endosomes with lysosomes, while those remaining in the limiting membrane can be recycled.^9^ Endosomal cargo recycling back to the plasma membrane can be routed via different pathways, for example, the small GTPases Rab4 and Rab35 regulate short‐loop or “fast” recycling from early endosomes, while cargoes recycled by the long‐loop or “slow” pathway are trafficked via the perinuclear endosomal recycling compartment (PNRC, also known as ERC) in a Rab11a‐ and Arf6‐dependent manner.^10^ In addition, retrieval of cargos from late endosome/lysosomes is an emerging mechanism controlling receptor recycling that is linked to cell migration and invasion.^11^

It is well established that receptor internalization is necessary to control the specificity, magnitude and duration of downstream signalling. Endosomes can function as signalling platforms where receptors elicit the same or different responses to those that occur at the plasma membrane. Endosomes have an appropriate pH to preclude ligand‐receptor dissociation and sequester these complexes from the proximity of phosphatases; both of these can lead to extended activation of the downstream signalling.^12^

Endocytic trafficking is particularly important in polarized cells, including epithelia (with apical and basolateral domains^13^) and migrating cells (with clearly established leading and trailing edges^14^). In migrating cells, the hypothesis that membrane and cargos might be internalized towards the rear of the cell and recycled at the front proved attractive,^15^ but recent evidence indicates that such trafficking may be spatially restricted towards the cell front^16^, ^17^, ^18^ and/or that long‐distance trafficking may occur from the cell front to the rear to aid retraction.^19^, ^20^ In the following sections, we will highlight the role that trafficking of distinct classes of cargoes has in establishing and/or maintaining polarity in motile cells with emphasis on migration in physiological matrix and in vivo.

## 3 RTK TRAFFICKING AND SIGNALLING IN CELL MIGRATION

RTKs are high‐affinity cell surface growth factor receptors with intrinsic, ligand‐mediated tyrosine kinase activity, and are known to regulate a diverse range of cellular functions. The endosomal trafficking of RTKs has been shown to influence cell migration in a variety of systems, including by contributing to the spatial and temporal control of downstream signalling.^21^

### 3.1 Localized trafficking and signalling in development

**Figure 1:**
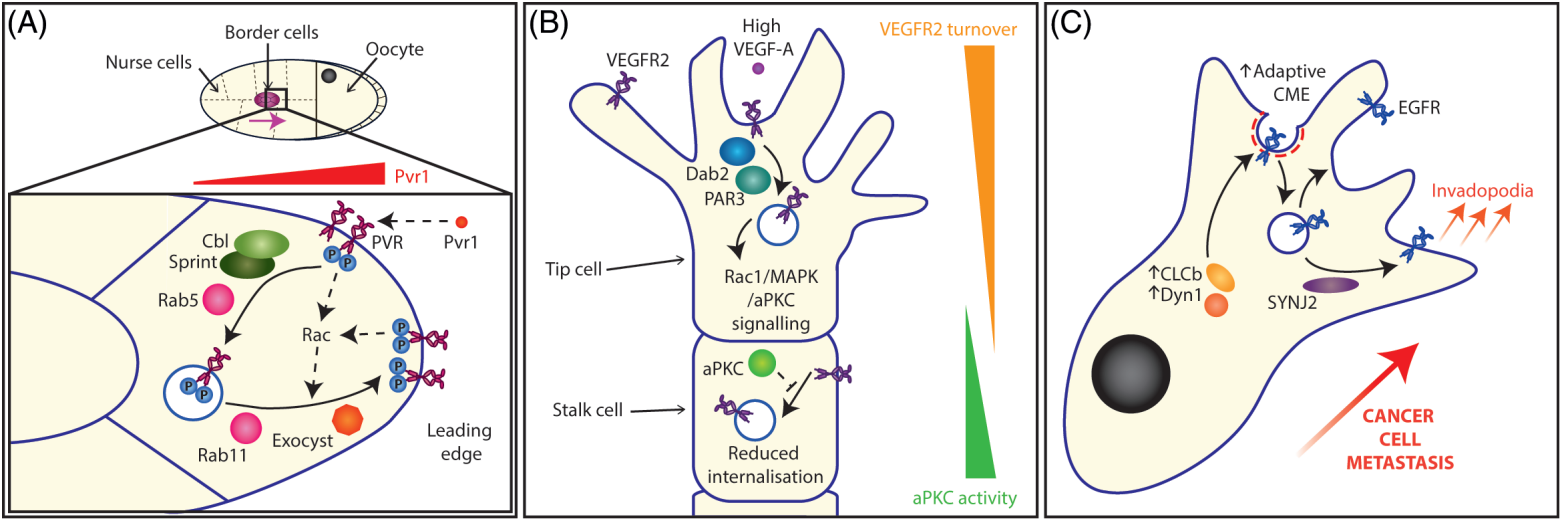
Receptor tyrosine kinase trafficking in cell migration. (A) In Drosophila melanogaster border cell migration PVR is internalized by Cbl, Sprint and Rab5, and subsequently recycled to the leading edge by Rab11 and exocyst. This leads to localized PVR signalling at the leading edge and drives collective cell migration. (B) In angiogenesis, VEGFR2 endocytosis occurs via Dab2 and PAR3 in migratory tip cells, which sustains Rac1, MAPK and aPKC signalling leading to cell migration. Meanwhile, VEGFR2 internalization is reduced in proliferative stalk cells due to the activity of aPKC. (C) Upregulation of CLCb and Dyn1 in cancer cells drives the adaptive CME of EGFR, thereby promoting EGFR signalling and leading to enhanced metastatic ability. SYNJ2 regulates EGFR recycling to the cell surface, driving invadopodia formation

In *Drosophila melanogaster*, border cells use chemotactic mechanisms to collectively migrate between nurse cells towards the oocyte during oogenesis. This requires localized signalling of the RTKs EGFR (epidermal growth factor receptor) and PVR (PDGFR‐ and VEGFR‐receptor related, the *D. melanogaster* PDGFR (platelet‐derived growth factor receptor)/VEGFR (vascular endothelial growth factor receptor) orthologue) at the leading edge.^16^ Local signals are maintained through an endocytic recycling loop, Cbl/Sprint/Rab5‐mediated receptor internalization followed by Rab11‐mediated local recycling and exocyst‐mediated delivery of these active receptors to the front of leader cells in the collectively migrating cluster.^16^, ^22^, ^23^ Interestingly, polarized signals are elicited by the PVR ligand Pvr1, and could involve positive feedback. PVR signalling promotes the localization of Rab11‐recycling endosomes to the leading edge through Rac signalling, which in turn supports the polarized distribution of PVR activation at the front of leader cells, promoting collective cell migration^24^ (Figure 1A).

Endocytic recycling also plays a key role in endothelial cell function (particularly through the recycling of integrin cargoes^25^, ^26^, ^27^, ^28^, ^29^, ^30^), and VEGFR2 trafficking is important in regulating angiogenic signalling.^31^, ^32^, ^33^, ^111^ During angiogenesis, sprouting endothelial cells are classified as either migratory tip cells or proliferative stalk cells, which respond differently to VEGF (vascular endothelial growth factor). Using postnatal vascularization of the mouse retina as a model system, a higher rate of VEGFR2 turnover was observed in tip cells compared with stalk cells, enabling a fast, strong and directional response upon ligand detection due to continual redistribution of both inactive and activated receptors.^33^ VEGFR2 endocytosis is mediated by the clathrin‐adaptor protein Dab2, and the polarity protein PAR3, which can contribute to polarized CME of integrins in 2D by directing protein kinase C (PKC)‐dependent phosphorylation,^34^ and is required to sustain Rac1, MAPK and atypical PKC (aPKC) signalling pathways^33^ (Figure 1B). Together this indicates that VEGFR2 trafficking is tightly regulated for precise signalling to drive specific cellular processes within different sprouting endothelial cell subtypes.

### 3.2 RTK trafficking and signalling in cancer

RTK trafficking, and the impact of this on signalling, has been implicated in cancer cell migration and metastasis.^35^ For example, upregulation of clathrin light chain b (CLCb) and dynamin‐1 (Dyn1) is correlated with poor prognosis in non‐small‐cell lung cancer. CLC1b and Dyn1 control the “adaptive” CME of EGFR, as opposed to constitutive CME governed by CLCa/b and Dyn2, promoting EGFR trafficking and signalling, and enhancing the metastatic ability of cancer cells in vivo^36^ (Figure 1C). EGFR recycling also plays a role in invasion and metastasis, and Synaptojanin‐2 (SYNJ2), an inositol 5‐phosphatase implicated in breast cancer progression, is a key regulator of EGFR recycling to promote the formation of lamellipodia, invadopodia and metastases in vivo^37^ (Figure 1C). Endosomal trafficking of the RTK c‐Met (also known as HGFR, hepatocyte growth factor receptor) via recycling endosomes controls the activation of Rac, and signalling to the cytoskeleton, to promote cancer cell migration and invasion.^38^ Knockdown of NHE5 (neurone‐enriched Na+/H+ exchange) increases the pH of recycling endosomes, inhibiting the recycling of the c‐MET to the plasma membrane, its delivery to the leading edge of cells and downstream signalling via Akt/ERK and Rac/Cdc42 leading to impaired directed cell migration and loss of polarity.^39^

**Figure 2:**
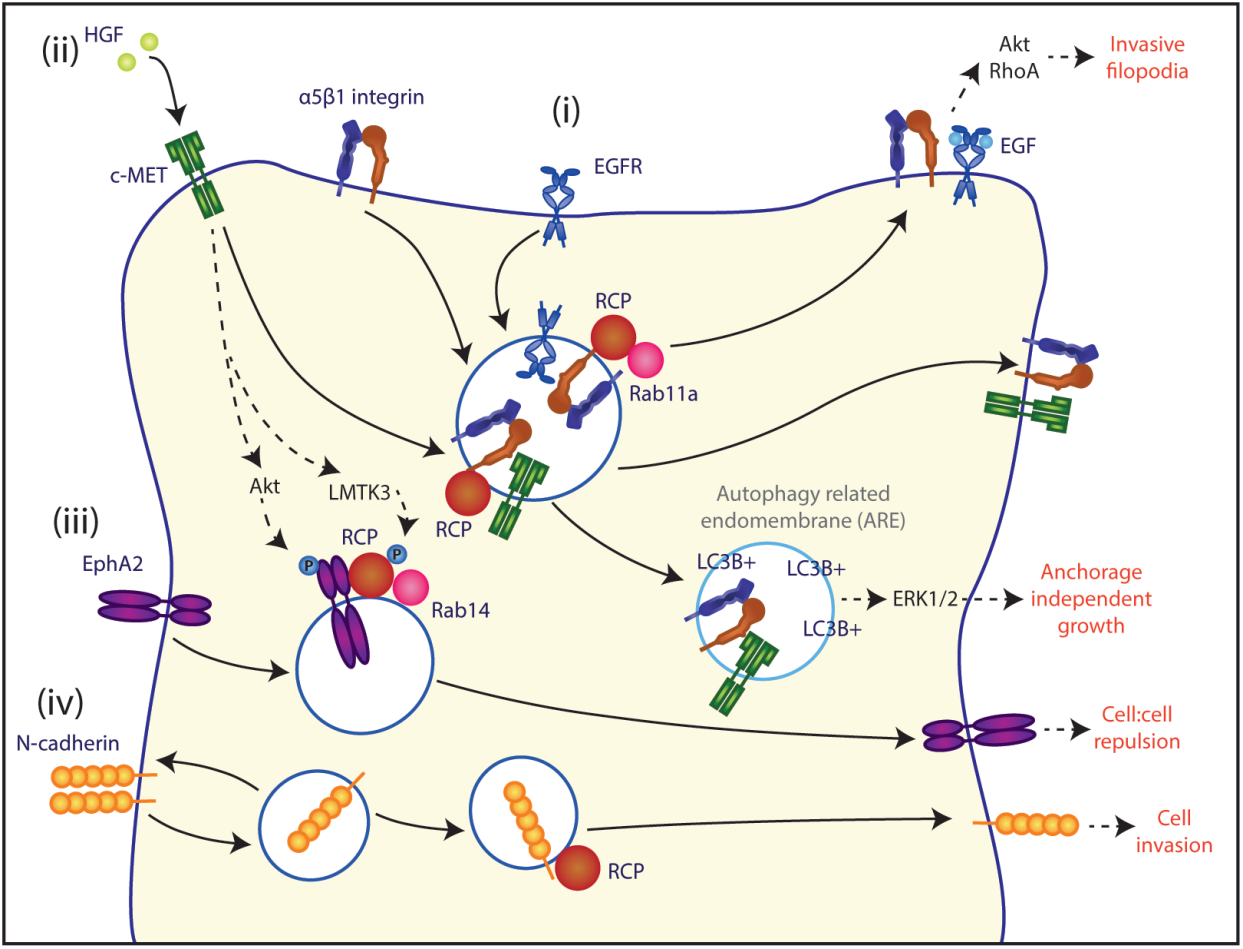
Rab coupling protein (RCP) in receptor recycling and co‐trafficking. (i) RCP controls co‐recycling of α5β1 integrin and RTKs to the leading edge, where RTK signalling is activated driving the formation of invasive filopodia. (ii) Upon HGF stimulation, c‐Met is co‐internalized with β1 integrin, and can follow an RCP recycling route to the cell surface. Co‐trafficking of c‐Met and β1 can also occur to LC3B‐positive autophagy‐related endomembranes, leading to sustained c‐Met signalling and ERK1/2 signalling. (iii) Trafficking of internalized EphA2 is regulated by RCP and Rab14. c‐Met signalling triggers LMTK3‐mediated phosphorylation of RCP, increasing its association with Rab14, and Akt‐mediated phosphorylation of EphA2, leading to cell:cell repulsion. (iv) N‐cadherin trafficking is controlled by RCP to promote cancer cell invasion

While the above examples demonstrate the outcomes of endosomal recycling of RTKs independently of other cargoes, it has been shown that the co‐trafficking of RTKs with adhesion receptors can also function to promote cell migration. Rab‐coupling protein (RCP; Rab11‐FIP1) drives invasive migration of cancer cells in 3D environments by forming a complex with α5β1 integrin, and subsequently recruiting RTKs for co‐recycling to the plasma membrane at the cell front^18^, ^40^, ^41^ (Figure 2). Here, enhanced recycling promotes RTK signalling to drive cell invasion, particularly by the proinvasive kinase Akt and a RhoA‐FHOD3 pathway promoting filopodia formation at the cell front.^42^, ^43^ Notably, FHOD3 knockdown does not impact on migration on 2D surfaces, but suppresses filopodial‐driven invasion in cell‐derived 3D matrix and 3D hydrogels.^43^ EphA2 is also a cargo of RCP, in this case directed by Rab14, and this trafficking pathway controls invasion and metastasis in pancreatic cancer^44^ (Figure 2). Additionally, hepatocyte growth factor (HGF) stimulation leads to co‐internalization of c‐Met (HFGR) and β1 integrin, and is required for downstream signalling in a variety of cell types,^45^ and indeed c‐Met can follow a RCP‐α5β1 recycling route to promote cancer cell invasion^41^ (Figure 2). Co‐trafficking of c‐Met and β1 integrin progresses to LC3B‐positive compartments that are part of a non‐canonical autophagy pathway and are referred to as autophagy‐related endomembranes (ARE). From ARE it is suggested that active β1 integrin acts as an adaptor between c‐Met and Shc, leading to sustained c‐Met signalling through ERK1/2. In this context, β1 integrin has been shown to be required for both anchorage‐independent growth in soft agar and c‐Met‐dependent in vivo invasion in zebrafish embryos^45^ (Figure 2). Coordinated endosomal trafficking of RTKs and integrins is therefore a key mechanism through which distinct receptor classes crosstalk to promote cell migration and invasion.

## 4 EXTRACELLULAR CHEMOTACTIC GRADIENTS IN DIRECTED CELL MIGRATION

Directed cell migration is driven by cells sensing and responding to external gradients of chemotactic factors.^46^ Major families of chemoattractants include soluble chemokines and growth factors. The efficiency of cell migration along a gradient is determined by both the responsiveness of a receptor to a chemoattractant and the levels of that receptor at the cell surface.^47^ Therefore receptor bioavailability, and hence cell migration, are regulated by endosomal trafficking.

Chemokine receptors can be internalized by clathrin‐dependent and ‐independent pathways, after which the fates of receptors and ligands may differ.^47^ In T lymphocytes, for example, CCR7 has been shown to undergo clathrin‐dependent endocytosis and recycling back to the plasma membrane, in order to drive directed cell migration, while its ligand, CCL19, is targeted for lysosomal degradation.^48^ Conversely, endocytosis of CXCR3 was found to be mediated by arrestins, independent of clathrin and caveolae, followed by receptor degradation rather than recycling.^49^

Chemokine receptor trafficking is fundamental to directed cell migration in vivo. A study in *Danio rerio* (zebrafish) embryos revealed a specific role for CXCR4 internalization and subsequent downregulation. CXCR4 internalization is dispensable for initial detection and response to the ligand SDF‐1a, but crucial for the fine‐tuning of cell migration to ensure correct directionality of primordial germ cells to the gonad development region.^50^ Moreover, studies of receptor CXCR7 and chemokine CXCL12 in the zebrafish lateral line primordium demonstrated that migrating cell collectives are capable of self‐generating chemokine gradients by polarizing receptor‐mediated internalization of ligands.^51^ This study provided the first in vivo evidence for self‐directed tissue migration driven by shaping an extracellular chemokine gradient.

## 5 REGULATION OF ADHESION BY RECEPTOR RECYCLING IN INVASIVE CELLS

Adhesion receptors, including receptors for ECM (eg, integrins, syndecans and discoidin domain receptors) and cell‐cell adhesion receptors (cadherins), play crucial roles in cell migration in physiological contexts. Trafficking of these receptor classes is important in controlling their localization at the cell surface, formation of cell‐matrix or cell‐cell adhesion complexes, and the signals generated downstream. Interestingly, crosstalk exists between receptor classes, for example, syndecan‐4 differentially regulates the trafficking of specific integrin heterodimers in migrating cells. Here, we will focus on the regulation and function of integrin trafficking in physiologically relevant 3D environments and in vivo, and highlight recent evidence for the involvement of cadherin trafficking in these processes.

Integrins are the primary adhesion receptors for components of the ECM, composed of non‐covalently linked α and β subunits that heterodimerize to form 24 distinct integrins expressed in a cell‐ and tissue‐specific manner.^52^ Integrin receptors have a large extracellular domain that binds to the ECM, and a short intracellular tail that interacts with a large number of cytoplasmic partners to link to the cytoskeleton or trafficking machinery. Upon association with the ECM, integrins cluster into dynamic complexes called focal adhesions, the components of which are collectively known as the “adhesome.”^53^ Adhesion complexes are able to transmit force to the actin cytoskeleton to regulate cellular processes, such as cell migration.^54^, ^55^ In order for cells to migrate in both 2D and 3D environments, focal adhesions must undergo disassembly and reassembly, and this is at least in part mediated by the trafficking of integrins.^56^, ^57^ Of note, integrin cytoplasmic tails frequently interact directly with regulators of endocytosis, endosomal sorting and endocytic recycling, an unusual feature of endocytic cargoes (see below).

**Figure 3:**
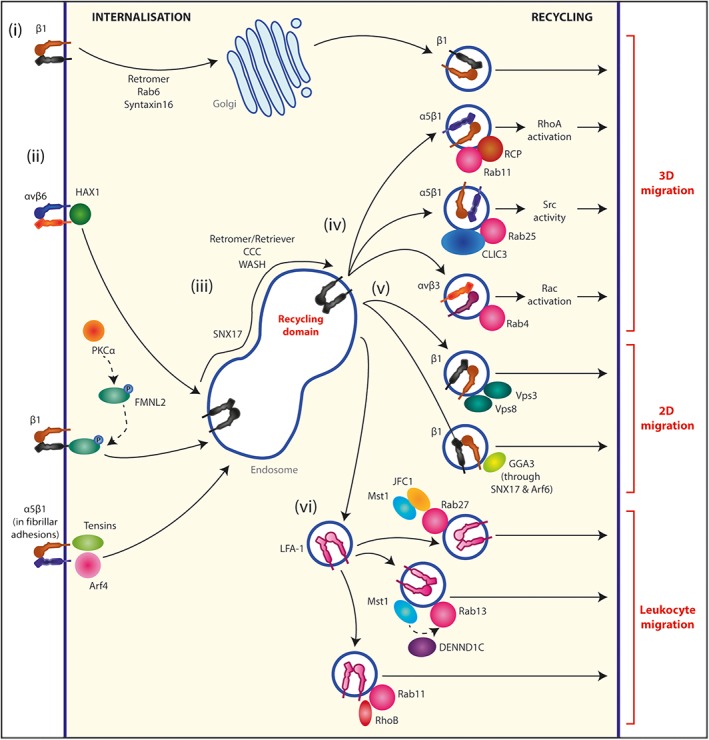
Integrin trafficking in cell migration. (i) Ligand‐free β1 integrins are trafficked through the retrograde pathway, from the plasma membrane to the TGN, controlled by retromer, Rab6 and syntaxin‐16. From the TGN integrins are recycled back to the plasma membrane where they contribute to 3D cell migration. (ii) Integrin internalization can regulate cell migration and occurs by many mechanisms; HAX1 controls αvβ6 endocytosis, phosphorylated FMNL2 controls β1 integrin endocytosis, Tensins 1‐3 and Arf4 control α5β1 endocytosis. (iii) Integrins are selected for recycling by SNX17, which drives the sorting of integrins into an endosomal “retrieval” domain. From here a relay of protein complexes sorts integrins into recycling domains. (iv) α5β1 integrins can be recycled via an RCP/Rab11a pathway or via a Rab25/CLIC3 pathway to drive cell migration in 3D environments. αvβ3 integrins can be recycled by a Rab4 pathway to drive lamellipodial‐driven 3D migration. (v) β1 integrin recycling can be controlled by both a Vps3/Vps8 route or a GGA3 route to drive 2D cell migration. (vi) Recycling of the integrin LFA‐1 controls the rapid motility of leucocytes. Mst1 associates with JFC1 to regulate Rab27‐mediated return of LFA‐1 to the cell surface. Mst1 also activates Rab13, via DENND1C, leading to LFA‐1 delivery to the leading edge. LFA‐1 can also be recycled via Rab11 vesicles, controlled by vesicle‐associated RhoB

It is now clear that focal adhesion complexes can form in cells migrating in 3D matrix^58^; however, how endocytosis might contribute to turnover in invasive cells is not known. Integrin internalization is required for cancer cell invasion, for example, HAX1 (hemopoietic specific protein‐1 [HS1]‐associating protein X‐1), a ubiquitously expressed protein, directly interacts with the cytoplasmic tail of β6 integrin to control internalization of αvβ6 integrin through a clathrin‐mediated pathway to drive cancer cell invasion^59^ (Figure 3). More recently, protein kinase C α (PKCα) has been shown to phosphorylate the formin FMNL2, releasing its auto‐inhibition and driving its rapid relocalization to endosomal membranes. FMNL2 interacts with the cytoplasmic tails of integrin α subunits to drive β1 integrin internalization and invasion of melanoma cells^60^ (Figure 3). Fibrillar adhesions are elongated subnuclear adhesions related to focal adhesions that are present in cells migrating on 2D surfaces. Tensins 1‐3 (components of fibrillar adhesions) and Arf4 coordinate endocytosis of α5β1 integrin and associated matrix proteins (eg, fibronectin) to late endosomes/lysosomes, where they contribute to nutrient sensing via mTOR (mammalian target of rapamycin) and cancer cell invasion^61^ (Figure 3).

### 5.1 Integrin recycling controls invasive cell migration

The rate of integrin degradation is low, and the majority of receptors are recycled back to the plasma membrane following endocytosis.^57^ The sorting events that control the selection of integrins for recycling over degradation became evident following the identification of sorting nexin 17 (SNX17) as a direct binding partner of the β1 integrin cytoplasmic tail.^62^, ^63^ SNX17 promotes the sorting of integrins into an endosomal “retrieval” domain (distinct from the ESCRT (endosomal sorting complexes required for transport)‐degradative domain), where a protein complex “relay” consisting of at least three protein complexes (Retriever, CCC and WASH) sorts integrins into recycling domains,^64^ which subsequently link to Rab‐dependent trafficking pathways via as yet unknown mechanisms (Figure 3).

There is now a wealth of evidence supporting a role for integrin recycling, particularly through Rab4 and Rab11 routes, in cell migration^57^ (Figure 3). Because of the subcellular distribution of these endosomes towards the leading edge of cells migrating in 3D matrix, integrin trafficking can provide a “spatially restricted” signal at the front of invading cells, with integrins and co‐cargo receptors internalized from, and recycled to, the same area of plasma membrane.^17^, ^18^, ^57^ Interestingly, Rab4 and Rab11 can handle different integrin cargoes, αvβ3 and α5β1 respectively, and there is a well‐documented antagonistic relationship between the fibronectin receptors αvβ3 integrin and α5β1 integrin. αvβ3 integrin activates Rac to promote slow and persistent migration, while α5β1 integrin activation of RhoA leads to rapid and random migration.^65^ Recycling of αvβ3 integrin via the “fast,” Rab4‐dependent recycling pathway requires the direct interaction of protein kinase D1 (PKD1) with the β3 integrin subunit, as well as PKD1‐dependent phosphorylation of Rabaptin‐5, to promote lamellipodia‐driven migration.^66^, ^67^, ^68^, ^69^ Disruption of this pathway by inhibiting αvβ3 integrin or through PKD1/Rabaptin‐5 mutation causes an increase in α5β1 integrin recruitment to RCP.^18^, ^69^ Increased “slow” Rab11‐ and RCP‐dependent α5β1 integrin recycling leads to the formation of ruffling protrusions for faster migration.^67^, ^68^, ^69^ In 3D environments the composition of the matrix is important in determining invasion: in low fibronectin 3D matrix (collagen or matrigel) αvβ3 integrin recycling promotes invasion; when fibronectin levels are high, however, αvβ3 recycling suppresses invasion, but inhibiting αvβ3 (or Rab4‐dependent recycling of αvβ3) promotes α5β1 integrin recycling to drive increased invasion.^18^, ^69^

Rab11/RCP‐dependent recycling of α5β1 is of particular interest in the context of cancer and metastasis‐promoting gain‐of‐function mutant p53 expression. Mutant p53 acts to suppress the ribonuclease Dicer and miR expression, and this in turn relieves an inhibition of RCP‐α5β1 association and consequently leads to α5β1 recycling.^40^, ^70^ As previously discussed, α5β1 integrin co‐traffics with RTKs in RCP‐containing vesicles. These are recruited by diacylglycerol kinase α (DGKα) production of phosphatidic acid at the tips of pseudopods invading into 3D ECM.^71^, ^72^ Localized RTK signalling initiates a signalling cascade that activates Akt, which then recruits a RacGAP1/IQGAP1 complex that inhibits Rac and subsequently increases RhoA activity at the cell front.^18^, ^42^, ^71^ RhoA activates the formin FHOD3, which promotes the nucleation of actin filaments and the formation of filopodial actin‐spike protrusions. These protrusions have been shown to mediate invasion in fibronectin‐rich 3D matrix in vitro, as well as in an in vivo zebrafish model.^43^ Furthermore, mathematical modelling of this signalling network has revealed that a MAPK‐driven feedback loop functions to maintain Rac inhibition, and that Rab11/RCP‐driven cancer cell invasion can be disrupted by MEK inhibition.^73^

α5β1 integrin recycling can also drive invasion into fibronectin‐rich environments by another mechanism. Rab25, a Rab11 family member with a more restricted expression profile, can directly bind the cytoplasmic tail of β1 integrin. This enables Rab25 to regulate the recycling of inactive α5β1 integrin back to the plasma membrane at the tips of migrating cells, for further interaction with the ECM to promote protrusion formation.^17^ In addition, active α5β1 integrin recycling from the cell front to the cell rear occurs via Rab25 endosomes and chloride intracellular channel 3 (CLIC3)‐positive lysosomes. Instead of being targeted for degradation, active α5β1 integrin receptors are rapidly recycled to the plasma membrane at the cell rear. Subsequent localized Src activity drives forward movement of the cell rear, further promoting invasion.^19^ This indicates that multiple trafficking pathways of a single integrin heterodimer can coordinate both protrusion formation and cell rear retraction, leading to the forward movement of the cell in 3D environments. Interestingly, Rab25 expression impacts upon 3D migration and invasion, but does not affect random migration on 2D surfaces.^17^

### 5.2 New pathways and machinery controlling integrin trafficking

More recently, a more detailed picture of the integrin trafficking machinery has been uncovered in cells migrating in 2D, including a Vps3 and Vps8 route that controls β1 integrin recycling, and how GGA3, an Arf6 effector, controls SNX17 localization to control motility in 2D (Figure 3). APPL1 has been shown to restrict cancer cell migration by modulating α5β1 trafficking, leading to increased presence at the cell surface, and decreasing Rac activity in a Rab5‐dependent manner.^74^ Recent studies have demonstrated that conformationally active integrins are found on endosomes and that integrin endosomal signalling, via focal adhesion kinase (FAK), can contribute to cancer‐related processes, including avoidance of anoikis, anchorage‐independent growth and experimental metastasis.^75^ Furthermore, the conformational memory of recycling integrins enables enhanced adhesion complex reassembly at the leading edge, in order to drive directional cell migration.^76^ These observations suggest a requirement for integrin signalling from endosomes to promote cell migration within 3D and in vivo contexts.

In addition to Rab4 and Rab11 pathways, new and unexpected trafficking routes have been identified. The retrograde trafficking pathway handles the delivery of cargos from the plasma membrane to the Golgi, and recent evidence demonstrates that ligand‐free β1 integrins follow this route in epithelial cells and fibroblasts. In this context, the retromer complex, Rab6 and syntaxin‐16 control the retrograde traffic and delivery of ligand‐free β1 integrins to the trans‐Golgi network (TGN), from where integrins return to the plasma membrane at the leading edge of polarized cells. This promotes directional migration in cells in 2D and directional migration of the distal tip cell along the basement membrane to form the gonad in *Caenorhabditis elegans* larvae^77^ (Figure 3).

### 5.3 Trafficking integrins in migrating leucocytes

Leucocytes are fast‐moving cells that have to quickly transmigrate from blood vessels into tissues upon signals of inflammation and infection. The major integrin used by leucocytes to achieve this is LFA‐1 and it provides an excellent model as to how integrin recycling can regulate cell motility. The intracellular trafficking of the integrin LFA‐1 has been highly studied as this can regulate cell adhesion and motility. For instance, it has been shown that upon T‐cell stimulation with chemokines, the kinase Mst1 activates Rab13 through the Rab13 GEF (guanine nucleotide exchange factor), DENND1C. This activation facilitates the delivery of LFA‐1 to the leading edge, whereby this spatial distribution of LFA can drive lymphocyte migration and trafficking in vivo^78^ (Figure 3). Mst1 has also been shown to associate with the Rab27 effector JFC1 (synaptotagmin‐like protein 1) regulating the trafficking of Rab27 vesicles back to the plasma membrane, further implicating Mst1 in controlling vesicle trafficking in migrating lymphocytes, and this pathway controls the ability of lymphocytes to cross intact basement membranes^79^ (Figure 3). Recently, vesicle‐associated RhoB has been shown to control Rab11 recycling of LFA‐1 to the cell surface along the microtubule network in migrating lymphocytes. T‐lymphocytes that lack functional RhoB exhibit reduced surface levels of LFA‐1, which leads to reduced T‐cell adhesion and migration mediated by the ligand ICAM‐1^80^ (Figure 3). This suggests that there may be common pathways that control integrin trafficking in adherent and non‐adherent cell populations.

### 5.4 Cadherins in cell migration and morphogenesis

Cadherins are the primary adhesion molecules that form cell‐to‐cell contacts called adherens junctions. Regulation of cadherins plays an essential role in physiological processes such as embryonic development, wound healing and cancer metastasis. Cadherins at the surface of adjacent cells connect via calcium‐dependent homophilic interactions between their extracellular domains. Intermediate proteins link the intracellular domain of cadherin to the actin cytoskeleton. Trafficking of cadherins has emerged as a fundamental mechanism by which these adhesive contacts can be regulated (expertly reviewed in References 2, 81).

Epithelial‐to‐mesenchymal transition (EMT) is involved in cell migration during development and disease.^82^ Since cadherins are the major components of epithelial adherens junctions, their removal from the cell surface is necessary for EMT to occur. EMTs enable cells to become more motile and leave the surrounding tissue. This is considered important for the initiation of cancer metastasis and invasive growth. The internalization and degradation of cadherins provides a rapid means by which to disassemble these contacts.^83^ Regulating the abundance of cadherin molecules at the plasma membrane has clear functional consequences, but vesicular pools of cadherin also have a role; for example, cadherin has been shown to co‐localize with active Rap1 in recycling compartments and drive EMT upon subsequent integrin activation.^84^ Unsurprisingly, cadherin trafficking does not occur in isolation but is coordinated with recycling pathways of other receptors. The cell surface levels of cadherins and integrins have been shown to be inversely modulated during cell migration. For example, Rab35 simultaneously promotes cadherin localization to the plasma membrane and inhibits Arf6, thereby downregulating recycling of β1 integrin subunits and EGFR.^85^ Analogous to its function in trafficking integrins, the Rab11 effector RCP controls N‐cadherin trafficking to promote invasion in lung cancer cells^86^ (Figure 2); in the future, it will be interesting to see if co‐regulation of different adhesion receptor classes is a feature of endocytic recycling pathways in invasion and metastasis.

Cadherin trafficking also plays a role in the maintenance of cell‐cell junctions during morphogenesis and collective migration, and logically endocytosis could contribute to cell‐cell adhesion plasticity to allow morphological changes to take place. Movements in convergent extension of the *Xenopus* animal cap are regulated by internalization of C‐cadherin, controlled by dynamin and Rnd1.^87^, ^88^ Furthermore, Wnt11 controls Rab5c‐dependent trafficking of E‐cadherins during zebrafish gastrulation.^89^ Interestingly, N‐cadherin is predominantly internalized at the rear of leader cells for recycling towards the front for reincorporation in adherens junctions in collectively migrating astrocytes,^90^ suggesting that endocytosis and targeted recycling may also be a key factor in collective movements. Given the burgeoning interest in collective migration, it seems likely that endocytic trafficking of cell‐adhesion molecules will become a focus of attention for this mode of movement in 3D and in vivo in the future.

## 6 CONTROL OF MATRIX PROTEOLYSIS BY ENDOCYTIC TRAFFICKING

Migration and invasion of individual cancer cells in 3D matrix and in vivo has been well studied, characterized and categorized into mesenchymal and amoeboid subtypes. Amoeboid migration is characterized by high levels of actomyosin contractility, which generates hydrostatic pressure to promote protrusion, and there is little protease activity required for cells to squeeze through gaps in the ECM.^91^ Mesenchymal migration, however, requires the concerted action of actin‐based protrusion and protease activity to allow migration through dense basement membrane matrix, which forms tissue boundaries, and fibrillar interstitial matrix.^91^, ^92^

While numerous proteases, membrane tethered and secreted, contribute to cancer cell invasion and metastasis, MT1‐MMP (membrane‐type‐1 matrix metalloprotease; MMP‐14) has become established as the major player in executing programmes of basement membrane and interstitial matrix invasion.^92^ MT1‐MMP is a transmembrane protein, and is hence subject to cycles of endocytosis and recycling that controls cell surface availability and function, perhaps by circumventing rapid inactivation by TIMP‐2 at the cells surface.^92^

Like other trafficking receptors, MT1‐MMP can follow several routes through the endocytic system. MT1‐MMP internalization is not well characterized but may occur via caveolae.^93^ Internalized MT1‐MMP reaches early endosomes before recycling via a number of different routes, which may depend on the specific cell/tissue type. MT1‐MMP is trafficked via microtubules,^94^ and phosphatidic acid is required for the recruitment of KIF5b to MT1‐MMP vesicles, which are delivered to the cell surface to promote invasion and metastasis in breast cancer.^95^ Rab5a is upregulated in breast cancer, and together with Rab4 controls the delivery of MT1‐MMP to the cell surface to promote invasion and metastasis, and the progression of ductal carcinoma in situ to invasive ductal carcinoma.^96^ In macrophages, Rab5a, Rab8a and Rab14 control the trafficking of MT1‐MMP (perhaps via the TGN) to promote motility in 3D collagen.^97^ Interestingly, a Rab8‐dependent trafficking route had previously been reported to handle MT1‐MMP in invading breast cancer cells,^98^ suggesting some level of conservation between these cell types.

While most trafficking receptors that reach late endosomes/lysosomes are degraded, some can be recycled from these compartments, including MT1‐MMP.^92^ In breast cancer cells, several regulatory steps have been shown to control rescue of cargos from late endosomes and lysosomes, delivery to the plasma membrane and invadopodia formation/invasion including: CLIC3,^99^ WASH (thought to control actin polymerization to mediate delivery of MT1‐MMP to invadopodia^100^), Arf6‐JIP3/4 (controlling directional traffic along microtubules^101^), Rab2A (which interacts with the late endosomal HOPS complex^102^), aPKC^103^ and the SNARE VAMP7.^104^ Interestingly, delivery of lysosomes to invadopodia in the *C. elegans* anchor cell is key to formation of invasive protrusions rich in the MMP ZMP‐1, a critical step in the programme of invasion across the basement membrane during development of the reproductive system.^105^ This could suggest that lysosomal delivery of cargoes including MMPs could form an evolutionarily conserved mechanism that controls invasion.

## 7 CONCLUSIONS

Here, we have given an overview of the recent advances that have improved our understanding of how endosomal trafficking of a range of receptors regulates cell migration within 3D and in vivo contexts. Several recent studies have demonstrated the breadth of coordination between receptor families; from RTK and integrin co‐traffic to the synergistic relationship between integrins and syndecans, it is becoming increasingly clear that trafficking of different receptors cannot be considered in isolation. Indeed, gaining a complete understanding of the cooperation between signalling and receptors is essential to fully comprehend many aspects of cell behaviour. The discovery that many of these receptors actively signal from endosomal compartments has created another avenue of research that is not well established, especially in terms of in vivo cell migration. Studies that decipher the molecular pathways driving cell migration are a useful precursor for understanding and potentially targeting this process in specific diseases.

Approaches to exploring endosomal trafficking in cell migration still rely heavily on biochemical and immunofluorescence experiments in 2D environments. Therefore, approaches in the future should endeavour to utilize the most physiologically relevant models (Box 1). In vivo models should be used where possible, alongside representative 3D systems with appropriate cell types and stimulatory factors, in which ECM composition and mechanical properties have been faithfully emulated. Use of improved imaging techniques, including various forms of super‐resolution microscopy, could also allow improved precision by pinpointing spatially restricted signalling events in these systems.

Recent studies that have used “omics” approaches to dissect receptor signalling have been very successful. For instance, an integrated multi‐layered proteomics approach was used successfully to decipher the selectivity of EGF and TGF‐α (transforming growth factor alpha) on EGFR fate. It showed that phosphorylation of Rab7 and RCP recruitment were switches for the opposing fates of EGFR recycling and degradation, and that this controlled downstream signalling and subsequently cell migration.^106^ Such studies highlight the power of using proteomic approaches to probe endosomal recycling processes and the downstream signalling responses. In addition to this, although some studies have used unbiased approaches to identify the regulators and the trafficking machinery of specific endocytic recycling processes, this could be expanded upon. These techniques would allow the identification of weak and transient complex components that play important regulatory roles yet have so far not been identified and studied.

## Supporting information


**Editorial Process**
