# Supplementary material for: Vesicle trafficking pathways that direct cell migration in 3D matrices and in vivo
Source: Traffic. 2018 Sep 10;19(12):899–909. doi: 10.1111/tra.12605 (PMC6282850; doi:10.1111/tra.12605)
Supplement: Supplementary file 1 — Editorial Process [file TRA-19-899-s001.pdf]

Title:	**Vesicle trafficking pathways that direct cell migration in 3D and *in vivo***
Authors:	Beverley J. Wilson, Jennifer L. Allen and Patrick T. Caswell
Article Type:	Review

Monitoring Editor Rytis Prekeris
Date Submitted 24 June 2018
Date for Decision 1 15 July 2018
Date Resubmitted 20 July 2018
Accepted 23 July 2018

Decision and Reviews

Dear Dr. Caswell,

Thank you for taking the time recently to write your review "Directing vesicle trafficking for cell migration in 3D and in vivo" for Traffic. I asked two colleagues who are experts in the field to review the paper and their verbatim comments are appended below. I share the view of the referees that this is a well-written and timely review that will be of interest to the readers of Traffic. However, both of the referees have recommended expanding the discussion in some places that I think will strengthen the review. Although I cannot accept this review at this time, I believe that you will find the revisions necessary to be straightforward and I look forward to receiving your revised manuscript in the near future. To expedite the review when you resubmit, I encourage you to include a letter that outlines how you have addressed each of the concerns.

Sincerely,

Rytis Prekeris, PhD
Guest Editor

Referee's Comments to the Authors

Referee: 1

Comments to the Author
Wilson et al (Caswell) Traffic 2018

It has been known for many years that the polarized trafficking of integrins, cadherins and receptors for chemoattractants or growth factors is important for directional cell migration under both homeostatic and pathogenic (i.e. tumor metastasis) conditions. This review summarizes what is currently known about how trafficking controls migration and describes some of the important mechanisms that regulate the trafficking of integrins, cadherins and signaling receptors. An interesting and important aspect of the review is its discussion of the role of trafficking in both 3-dimensional in vitro models and in model organisms.

Although the review is fairly comprehensive, there are several aspects of it that could be strengthened:

1. It is somewhat disappointing that no mention is made of polarity in the context of migration-related trafficking. Migrating cells are polarized along the front/rear axis and require the polarized insertion of both integrins and signaling receptors in the leading edge membrane. How is this directionality controlled?
2. It is sometimes unclear whether the authors are referring to a 2D or 3D model system when describing a regulatory

mechanism. In specific circumstances where the mechanisms differ, it would be useful to directly compare them. For example, are fibrillar adhesions (p6) present in cells migrating in 3D matrices?
3. Many of the descriptions of regulatory mechanisms are quite superficial. It is understood that a review of this nature could become bogged down in detail. However, in many instances the authors mention regulatory proteins without describing what they do, or how. For example, on p3 they mention that Rab11 supports activation of the Drosophila PDGFR/VEGFR receptor ortholog PVR, without describing how. Similarly, on p4 they mention that PAR3 modulates endocytosis of VEGFR during angiogenesis, but do not describe how.
4. A similar problem relates to the co-trafficking of c-Met and b1 integrin to LCB3-positive “autophagy-related endomembranes”. What does this mean? Are these true autophagosomes or simply endosomes that contain LC3B? And how does the presence of b1 integrin in these structures sustain c-Met signaling?
5. What is HAX1, and how does it control internalization of avb6 integrins?
6. The discussion of recycling from late endosomes/lysosomes should be expanded to include more detail. As the authors note, there are few examples of recycling from such late compartments and the topic is therefore worth exploring in more depth.

Minor points:
1. P8 – RTKs do not phosphorylate or activate Akt directly. They initiate a signaling cascade that leads to Akt activation.
2. GGA3 is not a regulator of Arf6, it is an effector.
3. Do conformationally active integrins stimulate anoikis, as suggested on p9?
4. A reference is needed for the roles of Rab5a, Rab8a and Rab14 in the control of MT1-MMP trafficking in macrophages.

Referee: 2

Comments to the Author
Comments to the Author:
Beverley et al., “Directing vesicle trafficking for cell migration in 3D and in vivo”

This review provides a comprehensive and well written account of the membrane trafficking pathways that play a role in transporting cell surface proteins that are involved in cell migration to and from the cell surface. The authors specifically discuss these pathways in the context of the more physiologically relevant 3D and in vivo modes of cell migration. This is a timely review that will be of interest to both specialists in the field and researchers who focus on other biological subjects. The most recent and relevant research has been covered and cited in this article.

Major Points:
1. The Title is a little confusing. A title such as “Vesicle trafficking pathways that direct cell migration in 3D and in vivo” might be more clear to the reader.
2. Control/controlling used four times in Synopsis.
3. The authors spend one paragraph in the Conclusion discussing models for studying cell migration. I believe that the Review would be enhanced if they expand on this and dedicate a section in the main body of the article on this topic, and discuss the in vitro and in vivo models that they believe provide the most robust and physiologically relevant answers.

Minor Points:
1. There is a superfluous ‘by’ in the first sentence of Endosomal Trafficking section.
2. Extra ‘role’ in last sentence of page 3. Also it should be VEGFR2 not VEFGR2.
3. Page 6, paragraph 2, sentence 2; “…..that interacts with a large number….”.
4. An extra “contribute to” in paragraph 3, page 8.

Author Rebuttal

Dear Lisa,

We are now submitting our revised review. We are pleased that the reviewers found our manuscript interesting and timely, and are grateful for their comments that help to clarify and improve it. We have highlighted the changes we

have made in yellow to make it easier to identify these changes, and include a point by point response below.

If you require any further information, please do not hesitate to contact me.
Yours faithfully,

Dr. Patrick Caswell

Reviewer 1:
1. It is somewhat disappointing that no mention is made of polarity in the context of migration-related trafficking. Migrating cells are polarized along the front/rear axis and require the polarized insertion of both integrins and signaling receptors in the leading edge membrane. How is this directionality controlled?
We aimed to structure the review by organising our discussion around different classes of receptor cargo, rather than 'function'. We had hope that the relevant pathways that influence polarity were clearly indicated and regret that this wasn't the case. We have included a paragraph to address this directly in general terms, and point to the more detailed information that follows (page 3).
2. It is sometimes unclear whether the authors are referring to a 2D or 3D model system when describing a regulatory mechanism. In specific circumstances where the mechanisms differ, it would be useful to directly compare them. For example, are fibrillar adhesions (p6) present in cells migrating in 3D matrices?
Our discussion of migration mainly focuses on 3D migration, and where an interesting or relevant study has been performed on 2D we mention this in the text. There are very few studies that that directly compare the requirement for a pathway in 2D versus 3D migration, but where this has been assessed we include a comment on this (Rab25 and FHOD3 are both required for 3D migration/invasion, but not 2D; page 5 and page 8).
3. Many of the descriptions of regulatory mechanisms are quite superficial. It is understood that a review of this nature could become bogged down in detail. However, in many instances the authors mention regulatory proteins without describing what they do, or how. For example, on p3 they mention that Rab11 supports activation of the Drosophila PDGFR/VEGFR receptor ortholog PVR, without describing how.
Clarified positive feedback mechanism of PVR recycling by rewording and hope this is now more clear (page 4).

Similarly, on p4 they mention that PAR3 modulates endocytosis of VEGFR during angiogenesis, but do not describe how.
The original paper does not go into exact detail, but we now include an improved description and relate to another publication in which PAR3 was shown to mediate polarised clathrin-mediated endocytosis (albeit in 2D and of a different cargo; page 4).

4. A similar problem relates to the co-trafficking of c-Met and b1 integrin to LCB3-positive "autophagy-related endomembranes". What does this mean? Are these true autophagosomes or simply endosomes that contain LC3B? And how does the presence of b1 integrin in these structures sustain c-Met signaling?
We have lengthened our discussion of this paper (the only paper to describe such structures) to include the mechanism proposed by the authors (page 5)
5. What is HAX1, and how does it control internalization of avb6 integrins?
We now explain this more clearly (page 7), although the precise mechanism is not known.
6. The discussion of recycling from late endosomes/lysosomes should be expanded to include more detail. As the authors note, there are few examples of recycling from such late compartments and the topic is therefore worth exploring in more depth.
As indicated in our response to point 1, we tried to roganise the review by discussing classes of receptor, rather than specific trafficking route. We've included a sentence that we hope highlights recycling from LEs/lysosomes, and refer to a recent review that discusses this in more detail.

Minor points:
1. P8 – RTKs do not phosphorylate or activate Akt directly. They initiate a signaling cascade that leads to Akt activation. Changed as suggested (page 7).
2. GGA3 is not a regulator of Arf6, it is an effector. This mistake has been corrected, we're grateful that the reviewer spotted this.
3. Do conformationally active integrins stimulate anoikis, as suggested on p9? This is a mistake, we have changed this to 'avoidance of anoikis' as show by Alanko et al. We thank the reviewer for spotting this.
4. A reference is needed for the roles of Rab5a, Rab8a and Rab14 in the control of MT1-MMP trafficking in macrophages. Reference added.

Referee2:

Major Points:

1. The Title is a little confusing. A title such as “Vesicle trafficking pathways that direct cell migration in 3D and in vivo” might be more clear to the reader.

We are glad the reviewer pointed this out and have changed the title as suggested so that our meaning is clear.

2. Control/controlling used four times in Synopsis. We’ve re-written this section to avoid repetition.

3. The authors spend one paragraph in the Conclusion discussing models for studying cell migration. I believe that the Review would be enhanced if they expand on this and dedicate a section in the main body of the article on this topic, and discuss the in vitro and in vivo models that they believe provide the most robust and physiologically relevant answers.

We have included a box to discuss different types of 2D and 3D migration assay and their advantages and disadvantages. For in vivo models, we felt that these are too numerous to discuss and hope the explanation of these we have given in the main text is adequate to describe them.

Minor Points:

1. There is a superfluous ‘by’ in the first sentence of Endosomal Trafficking section. Corrected.
2. Extra ‘role’ in last sentence of page 3. Also it should be VEGFR2 not VEFGR2. Corrected.
3. Page 6, paragraph 2, sentence 2; “…..that interacts with a large number….”. Changed ‘that’ to ‘which’.
4. An extra “contribute to” in paragraph 3, page 8. Corrected.
